# Evaluation of vaginal ampicillin administration in late gestation sows on pelvic organ prolapse in relation to perineal score and litter characteristics

**DOI:** 10.1093/tas/txag153

**Published:** 2026-10-06

**Authors:** Zoë E Kiefer, Erika M Johnson, Jamie M Studer, Jason W Ross

**Affiliations:** Department of Animal Science, Iowa State University, Ames, IA, United States; Department of Animal Science, Iowa State University, Ames, IA, United States; Department of Animal Science, Iowa State University, Ames, IA, United States; Department of Animal Science, Iowa State University, Ames, IA, United States

**Keywords:** ampicillin, mitigation, piglets, pelvic organ prolapse, sow

## Abstract

Within the last decade, increased sow mortality resulting from pelvic organ prolapse (POP) has become a growing concern in the swine industry. Despite being widely acknowledged as a sow welfare and production issue, mitigation strategies are scarce. Previous studies have demonstrated differences in the vaginal microbiome are associated with risk for POP during late gestation, representing a potential target for prevention strategies. The objective of the current study was to evaluate effects of vaginal infusion of ampicillin during late gestation on POP incidence. To accomplish this, the hypothesis that vaginal infusions of antibiotics would reduce POP risk during late gestation was tested. Ampicillin was infused into the vagina at two timepoints during late gestation. The first dose (10 mL at 200 mg/mL) was administered at gestational day 96 ± 0.09 followed by a second, equivalent dose at gestational day 112 ± 0.10. Sows were randomly assigned to treated (TRT; *n* = 824) and non-treated (CON; *n* = 739) groups based on gestation pen. At gestational days 107 to 116, sows (*n* = 1076) were evaluated for POP risk using a three-point perineal scoring system. Briefly, a perineal score (PS) of 1 (PS1) presumes little to no risk of POP, and a PS of 3 (PS3) presumes high risk of POP. From the 1076 sows assigned a PS, 46.5%, 49.7% and 3.8% of sows were assigned PS1, PS2, or PS3, respectively. Ampicillin treatment had no effect on PS (*P* = 0.20), with 45.6%, 51.6% and 2.8% of CON sows and 47.2%, 48.1% and 4.7% of TRT sows scoring a PS1, PS2, or PS3, respectively. Additionally, treatment did not affect (*P* = 0.74) POP incidence, with 4.5% of CON sows and 4.7% of TRT sows subsequently experiencing POP. Ampicillin treatment did not affect (*P* ≥ 0.54) litter characteristics. However, there was an effect of PS on litter characteristics observed with total born and stillborn piglets (*P* < 0.01), and a tendency for a difference in liveborn piglets (*P* = 0.09) between sows scored PS3 and PS1. Sows assigned a PS3 had fewer piglets born alive, presumably due to the increased number of stillborn piglets. Additionally, PS3 sows had 1 more total born on average within their litters compared to PS1 assigned sows (*P* < 0.01). In conclusion, vaginal infusion of ampicillin had no effect on PS, POP incidence, or litter characteristics, but PS did have an effect on the number of piglets born alive.

## Teaser

Pelvic organ prolapse (POP) represents approximately 21% of sow mortality, and currently no established mitigation strategies exist to prevent POP incidence. The study conducted herein represents an important step in developing and testing approaches to reduce POP in late gestation sows.

## Introduction

Over the last decade, sow mortality has continued to rise despite the increase in reproductive performance. In 2023, the average sow mortality in the U.S. was 15.8%, a 6.9% increase from 2014 (Eckberg 2023). Pelvic organ prolapse (POP) makes up a substantial portion of this increase in mortality. A survey completed in 2018 (Ross 2019) evaluated causes of sow mortality across the U.S. and observed that over 21% could be contributed to POP. More recent studies have reported that POP accounts for approximately 28% of sow mortality, a proportion comparable to lameness and second only to sudden death or mortality of unknown cause (Paiva et al. 2023). This phenomenon is an industry wide issue and has been a research focus for the last 10 years due to the limited knowledge of underlying causes. Sows most commonly experience POP during the period leading up to, during, and shortly after farrowing (Supakorn et al. 2017). The losses due to POP in sows are not only an animal welfare concern, but an economic issue, which warrants the exploration and development of effective mitigation strategies.

Microorganisms typically participate in a symbiotic relationship with their host and have the ability to influence host health. In humans, dysbiosis of microbiota in the female reproductive tract has been linked to pelvic inflammatory disease (PID) and bacterial vaginosis (BV) (Eschenbach et al. 1975; Spiegel 1991; Green et al. 2015). Because of these observations in women, our group has begun to characterize the vaginal microbiota of late gestation sows and its association with POP. These studies have reported differences in the vaginal microbiota of late gestation sows that are at either low or high risk for POP (Kiefer et al. 2021a, 2021b, 2024). Specifically, increased abundance of *Streptococcus, Corynebacterium* and *Clostridium* were observed in vaginal microbiota of sows with increased risk for POP (Kiefer et al. 2021a, 2021b). Additionally, our group evaluated the vaginal microbiome metagenome and found virulence factors in *Streptococcus* and *Staphylococcus* (Kiefer et al. 2026). Within the gene catalog created from metagenomic sequencing of the vaginal microbiome of sows, several genes were observed that had the potential to have negative impacts on reproductive health (Kiefer et al. 2026). Additionally, fecal microbiota was evaluated in relation to POP, but fewer differences were observed compared to the vaginal microbiota (Kiefer et al. 2024). Collectively, these results suggest that the vaginal microbiota has greater influence on POP risk than the fecal microbiota. One component of many gram-negative bacteria is lipopolysaccharide (LPS), which can trigger an immune response causing the liver to release LPS binding protein (LBP) (Farhana and Khan 2023). Increased LBP in serum of sows at increased risk for POP has also been observed (Kiefer et al. 2021c). This led us to investigate if strategies to alter the vaginal microbiota may be a potential mitigation strategy for POP in late gestation sows.

Utilization of antibiotic pulses in the feed of sows has been associated with decreased mortality in sow farms (Ross 2019). Previous work evaluating bacitracin methylene disalicylate (BMD) as a POP mitigation strategy observed no difference in POP outcome (Studer et al. 2026). While oral antibiotic administration is common, the differences in vaginal microbiota in relation to POP led us to focus on an intravaginal delivery approach. In human medicine, intravaginal treatments are commonly used to treat BV (antibiotic) (Abbe and Mitchell 2023), and yeast infections (antifungal) (Watson et al. 2002). When evaluating antibiotics used within the swine industry, ampicillin was chosen to evaluate as a mitigation strategy because of its effect on specific genre of microorganisms (Peechakara et al. 2023). Specifically, ampicillin has been used to treat infections from *Streptococcus, Corynebacterium* and *Clostridium*, all of which were observed in higher abundance within the vaginal microbiota of sows at increased risk for POP previously (Kiefer et al. 2021a, 2021b). Additionally, *Streptococcus* and *Clostridium* were also observed to be increased in the fecal microbiota of sows at presumed higher risk for POP (Kiefer et al. 2024). Sows at high risk for POP can be identified as early as gestation week 12 (Kiefer et al. 2021b, 2021c) indicating a need for mitigation before the peripartum period when POP occurs. The objective of the current study was to evaluate the effects of vaginal infusion of ampicillin on POP risk and incidence rate. To do so, we tested the hypothesis that the use of intravaginally delivered antibiotics would reduce POP risk during late gestation.

## Materials and methods

### Animals and treatments

In total, 1563 sows were included in this trial. All experiments involving animals were approved by the Iowa State University (ISU) Institutional Animal Care and Use Committee. This work was conducted on a commercial sow farm over the course of 10 consecutive weeks during the winter season. Sows were housed in open pens during gestation, and animals in sick pens were excluded from the study. Treatments were randomly assigned and of the 1563 sows enrolled in the study, 739 sows were assigned to the control group (CON, no infusions) and 824 sows were assigned to the treated group (TRT, vaginal infusions of ampicillin). The goal was to reduce POP incidence by at least 1% in sows in the TRT group compared to CON and a power test was performed to determine the minimum number of animals needed.

### Vaginal infusions

Infusions of 10 mL of ampicillin (200 mg/mL) were given intravaginally using an Allflex Heavy-Duty Drencher with Backpack (CAT: C05227, Nasco Education Fort Atkinson WI, USA) with a 5in nozzle attachment. Dose was calculated using the recommended 2 mL per 100lbs body weight for intramuscular injections and estimating sow weight to be 500 lbs. Infusions were given at two timepoints prior to farrowing targeting application at approximately 3 weeks and again 3 days prior to expected farrowing. Timepoint 1 (TP1) infusions were given at gestational day 96 ± 0.09, and timepoint 2 (TP2) infusions were given at gestational day 112 ± 0.10.

### Perineal scoring system

Three perineal score (PS) classes, varying from presumed low to high risk, were used to classify sows (*n* = 1076) for POP risk during gestational days 107–116, as previously described (Kiefer et al. 2021a). For consistency, sows were only assigned a PS while lying down on specific days. Due to this only a portion of the total sows enrolled in the study were able to be assigned PS because sows who remained standing were unable to be assigned a PS. In brief, to assign the PS, the perineal region was visually evaluated for swelling, redness, and protrusion. A PS1 sow was considered little to no risk for POP, where a PS3 sow was considered to be at high risk of subsequently experiencing POP.

### Statistical analysis

The main effects of PS, Treatment, and POP were assessed in SAS 9.4 (Cary, NC) utilizing a PROC MIXED analysis. The effect of PS and Treatment on POP were assessed utilizing a PROC FREQ analysis. Additionally, PS and POP were analyzed by number of infusions using the previously mentioned methods. PROC FREQ was also used to analyze mortality between treatment groups. Litter characteristic data was analyzed using PROC MIXED evaluating the effects of Treatment and PS with parity as a covariant and breed week as a random effect. Data are considered significant if *P* ≤ 0.05 and indicate a tendency for biological meaning if 0.05 < *P* < 0.10.

## Results

### Animals

In total, 1563 sows were enrolled in the study with 739 sows assigned control (CON) and 824 sows assigned to the vaginal infusion treatment (TRT). Of the 824 sows in the TRT group, 87% received both infusions prior to farrowing. Some sows did not receive a second infusion due to farrowing prior to receiving the second dose. During the duration of the study, of the 1563 sows enrolled, 101 died. Of the sows that died, 43% were due to POP, 18% were due to sudden death, and 10% were due to lameness (Fig. 1). Treatment group had no effect on mortality rates (*P* = 0.92). Treatment group was not affected (*P* = 0.94) by parity with both CON and TRT sows having an average parity of 4.2 ± 0.10. Additionally, parity had no effect (*P* = 0.29) on PS.

**Figure 1:**
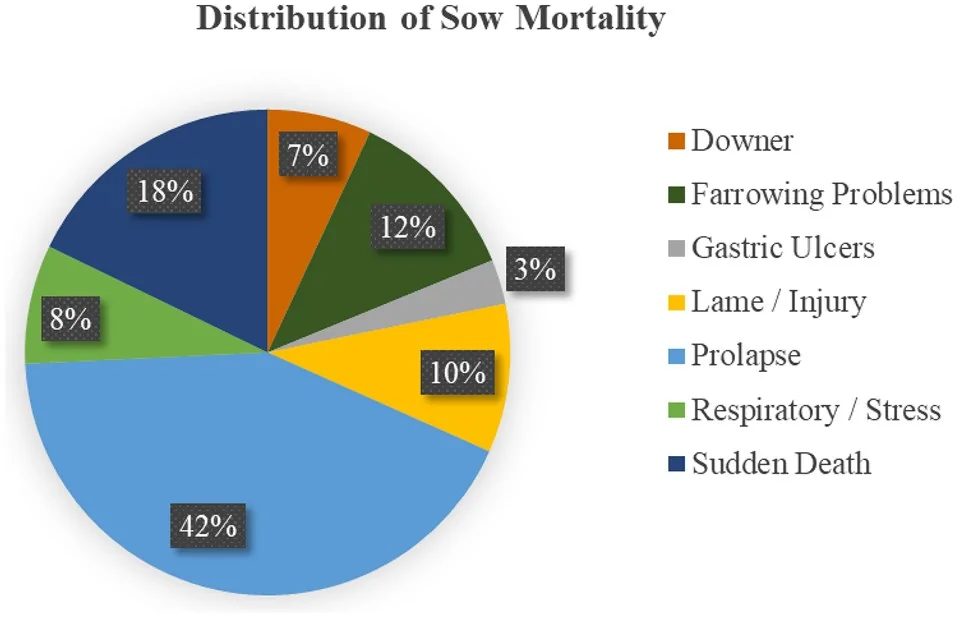
Distribution of sow mortality during project duration. Sow deaths (*n* = 101) for all sows enrolled in the study (*n* = 1563) were categorized into 7 different causes. These causes included downer (non-ambulatory), farrowing problems, gastric ulcers, lame, prolapse, respiratory/stress, and sudden death. This data only represents the time period that the trial was going on, and only includes the sows that were on trial. Pelvic organ prolapse (prolapse, light blue) represented 42% of all sow mortality.

### Effect of treatment on perineal score, prolapse outcome, and litter characteristics

In total, 1076 sows were assigned a PS during gestational day 114 ± 0.06. As previously demonstrated (Kiefer et al. 2021a, 2021b, 2021c, 2024), sows with a PS3 have a greater (*P* = 0.05) probability of experiencing POP. In total, 498 and 578 sows were assigned a PS from the CON or TRT groups, respectively. In the current study, 9.8% of PS3 sows subsequently experienced POP compared to 5.6% of PS2 sows and 3.2% of PS1 sows (Fig. 2). Antibiotic treatment via vaginal infusion did not affect PS (*P* = 0.21) with 45.6%, 51.6%, and 2.8% of CON sows and 47.2%, 48.1% and 4.7% of TRT sows being assigned PS1, PS2 and PS3, respectively (Fig. 3). Additionally, there was no effect (*P* = 0.58) of treatment on POP outcome with 4.4% and 4.8% of CON and TRT sows, respectively, subsequently experiencing POP. When evaluating outcomes for sows that received both infusions compared to those that only received one, no differences in POP outcome (*P* = 0.60) were detected.

**Figure 2:**
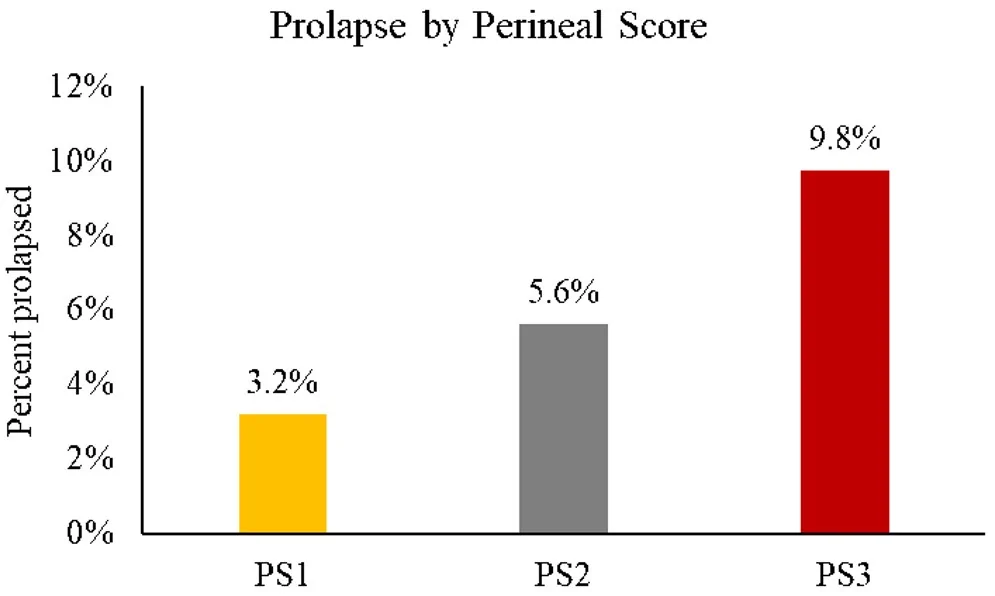
Distribution of pelvic organ prolapse by perineal score. A total of 1076 sows were assigned a perineal score (PS) during gestational day 114 ± 0.06. In brief, a sow assigned PS1 is presumed little to no risk for pelvic organ prolapse (POP), and PS3 is presumed high risk for POP. A PS3 sow is at significantly (*P* ≤ 0.05) higher risk for POP than a PS1 or PS2 assigned sow. The current study observed POP rates of 3.2%, 5.6%, and 9.8% of PS1 (*n* = 500), PS2 (*n* = 535), and PS3 (*n* = 41) assigned sows, respectively.

**Figure 3:**
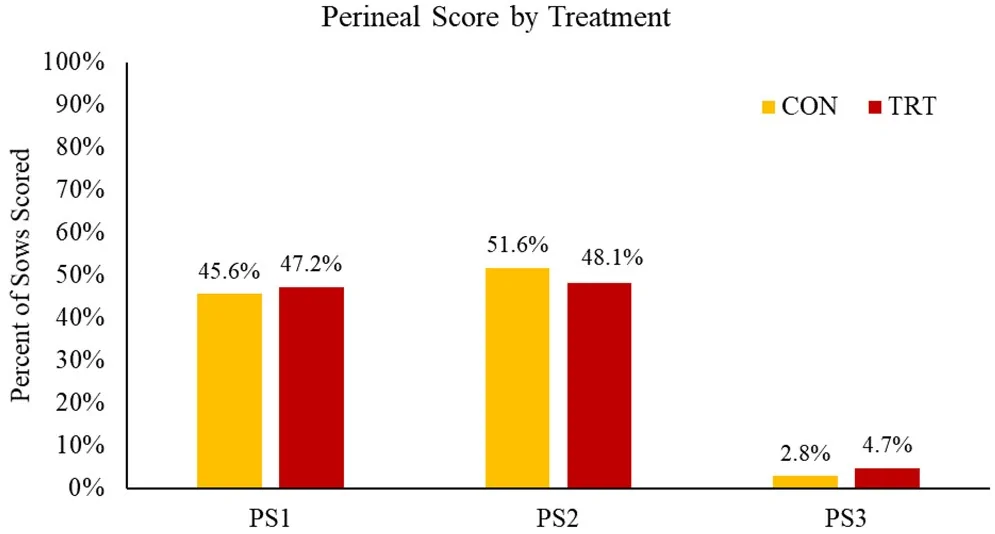
Treatment did not affect perineal score in late gestation sows. Vaginal infusions of ampicillin did not affect (*P* = 0.21) perineal score (PS) of late gestation sows. Briefly, a PS1 assigned sows is at low risk for pelvic organ prolapse (POP), and a PS3 assigned sow is at high risk for POP. Percentage of control sows (CON, *n* = 498) assigned PS1 (*n* = 227), PS2 (*n* = 273), or PS3 (*n* = 14), respectively, are represented by the yellow bars. Percentage of treated sows (TRT, *n* = 578) assigned PS1 (*n* = 273), PS2 (*n* = 278), or PS3 (*n* = 27), respectively, are represented by the red bars.

Treatment had no effect (*P* ≥ 0.36) on litter characteristics such as total born, number born alive, and stillborn. Additionally, litter characteristics were not affected by the interaction of PS and treatment (*P* ≥ 0.32). Mummies were not taken into consideration for this study as they were not recorded on an individual sow basis. Litter characteristic data by PS can be found in Table 1. Differences (*P* < 0.01) in total born were observed between PS which is likely driven by PS2 assigned sows having more total born than PS1 assigned sows (*P* < 0.01), as no differences were observed between PS1 and PS3 sows (*P* = 0.17) or PS2 and PS3 sows (*P* = 0.94). Litters from sows assigned PS3 (*n* = 38) had an average of 15.7 ± 0.90 total born piglets, with 12.8 ± 0.59 born alive, and 2.3 ± 0.30 stillborn. Sows assigned PS1 had litters (*n* = 489) with an average of 14.8 ± 0.66 total born piglets, with 13.3 ± 0.16 born alive, and 1.3 ± 0.08 stillborn. Litters from PS2 assigned sows (*n* = 523) had an average of 15.6 ± 0.66 total born piglets, with 13.7 ± 0.16 born alive, and 1.6 ± 0.08 stillborn. A tendency (*P* = 0.09) for differences in number of piglets born alive was observed between PS, with PS3 assigned sows having fewer piglets born alive compared to sows assigned PS1 and PS2. Additionally, stillborn piglets were higher (*P* < 0.01) in PS3 assigned sows compared to PS1 and PS2 (Table 1). When evaluating born alive and stillborn piglets as a percentage of total born piglets, differences (*P* < 0.01) were observed between sows assigned different PS. Live born piglets represented 90.6% ± 3.0%, 88.3% ± 3.0%, 82.9% ± 3.7% of total born for PS1, PS2 and PS3 sows, while stillborn piglets represented 8.0% ± 0.5%, 10.0% ± 0.5%, and 13.9% ± 1.9% of total born for PS1, PS2 and PS3 sows, respectively (Table 1).

**Table 1:** Evaluation of litter characteristics by perineal score.

Key performance indicators	Perineal score^1^				
	PS1	PS2	PS3	SEM	*P-*value
**Total born**	14.8^a^	15.6^b^	15.7^a,b^	0.90	< 0.01
**Live born**	13.3^x^	13.7^y^	12.8^x,y^	0.60	0.09
**% of Total born**	90.6^a^	88.3^b^	82.9^c^	3.73	< 0.01
**Stillborn**	1.3^a^	1.6^b^	2.3^c^	0.30	< 0.01
**% of Total born**	8.0^a^	10.0^b^	13.9^c^	1.89	< 0.01

1 Perineal Score (PS) of a 1 is considered little no risk for pelvic organ prolapse (POP) (*n* = 489 litters), a PS2 is considered moderate risk for POP (*n* = 523 litters), and a PS3 is considered high risk for POP (*n* = 38 litters).

a,b,c Signifies significance of *P* < 0.05.

x,y Signifies a tendency for significance 0.05 < *P* < 0.10.

## Discussion

The majority of sow mortality can be attributed to three primary categories: sudden or unknown death, lameness, and POP. Approximately 21% of sow mortality is attributed to POP (Ross 2019). Previous research has demonstrated that PS is indicative of POP risk, with PS3 sows experiencing POP at a substantially greater frequency than PS1 sows. Across studies, POP incidence has ranged from 7.2% to 23.4% in PS3 sows compared with 1.1% to 2.1% in PS1 sows (Ross 2019; Kiefer et al. 2021a, 2021b, 2021c, 2024; Studer et al. 2026). Although variability in POP incidence among PS categories has been reported across studies, higher PS has consistently been associated with greater POP incidence. This variability may be influenced by factors such as differences among individuals assigning PS or variation in farm POP incidence rates. The 9.8% POP incidence observed among PS3 sows in the current study falls within the expected range previously reported.

Previous work has reported differences in the vaginal microbiota of late gestation sows at various risk for POP (Kiefer et al. 2021a, 2021b), with fewer differences observed in the fecal microbiota (Kiefer et al. 2024). Thus, we explored the strategy of evaluating intravaginal infusion of antibiotics to mitigate POP and reduce POP risk as assessed by PS. Based on the data in this study, vaginal infusions of ampicillin during late gestation had no effect on POP risk or incidence.

In women suffering from BV and other reproductive diseases linked to vaginal dysbiosis, a common treatment is antibiotics. These antibiotic treatments can be given orally or in some cases, a vaginal infusion is utilized. Interestingly, in women the antibiotic administration results in changes in the vaginal microbiota and has been successful in treating disease (Mayer et al. 2015; Patangia et al. 2022; Kesavelu and Jog 2023). However, the current study is limited as the vaginal microbiota of sows was not evaluated due to the lack of an effective treatment outcome. Thus, understanding the effectiveness of the infusions in altering the vaginal microbiota in sows is not known.

Research evaluating the use of in-feed antibiotic pulses to reduce POP incidence has produced variable results. An industry survey reported reduced POP incidence during antibiotic administration and for a short period following antibiotic withdrawal, although analysis of yearly data did not identify a significant effect of antibiotic pulses on overall POP incidence (Ross 2019). The survey also identified a seasonal pattern in POP incidence, with greater incidence during winter compared with summer months (Ross 2019). It is possible that antibiotic pulses in the feed had a larger impact in the winter months, when POP occurrence is elevated. Further evaluations will be needed to understand the effect of antibiotic pulses in feed on POP incidence while taking into consideration the effect of seasonality.

Higher abundances of *Streptococcus, Corynebacterium*, and *Clostridium* populations were previously observed in sows assigned PS3 during late gestation compared to sows assigned PS1 (Kiefer et al. 2021a, 2021b), and known virulence factors are present in the vaginal metagenome (Kiefer et al. 2026). Ampicillin can be used to manage or treat infections caused by these microorganisms (Peechakara et al. 2023). In the current study, vaginal infusions of ampicillin did not affect PS or POP outcome, failing to support our hypothesis that the use of intravaginally delivered antibiotics would reduce POP risk during late gestation. There may be several potential reasons for this outcome. First, the timing of treatment could have affected success. Previous work has reported that gestation week 12 was the earliest a PS3 sow could be identified (Kiefer et al. 2021b, 2021c). Therefore, the current study focused on implementing a treatment approximately 3 weeks pre-farrow, however, that timing may not have given beneficial bacteria enough time to repopulate and establish a healthy microflora prior to the onset of POP around farrowing. Antibiotics can begin killing bacteria within 24 hours of administration, however it can take weeks or even years for the microbial population to return to normal (Patangia et al. 2022; Kesavelu and Jog 2023). It is possible that implementation of a strategy to alter the vaginal microbiota earlier in gestation in order to allow for the microbial population to return to a healthy state and repopulate could be beneficial to sow health. Additionally, strategies to positively impact and support a healthy vaginal microflora, such as the use of probiotics targeted to influence the vaginal microflora, may be an area of exploration. Alternatively, a combination treatment of antibiotics and subsequent probiotics could be used to kill off pathogenic bacteria and then allow for a quicker repopulation of beneficial bacteria. In humans, the use of a probiotic to help maintain healthy vaginal microbiota, or used as a treatment for diseases, has been observed to be beneficial (Cribby et al. 2008; Pendharkar et al. 2015; Lehtoranta et al. 2022).

Ongoing research seeks to define the composition and characteristics of a healthy vaginal microbiota in sows (Wang et al. 2017; Sanglard et al. 2020; Zhang et al. 2021; Poor et al. 2022). Additionally, the composition of a healthy vaginal microbiota is likely influenced by the sow’s reproductive status, suggesting that microbial communities may differ across physiological stages. Early gestation may differ from late gestation microbial communities, and understanding the differences in vaginal microbiota at different reproductive stages is a critical step towards understanding which microbes could be considered harmful to reproductive health in sows. Determining which bacteria, or combination of bacteria, may contribute to increased POP risk is important, in addition to determining causation of microbiota with reproductive dysfunction in sows. The current study focuses on decreasing specific genera of bacteria susceptible to ampicillin that have been previously observed to be higher in sows at high risk for POP (Kiefer et al. 2021a, 2021b). A limitation of this study is that characterization of the vaginal microbial community following vaginal ampicillin infusion was not performed, precluding evaluation of the treatment’s effects on the vaginal microbiota. These analyses were outside the scope of the study, as the primary objective was to evaluate the effects of ampicillin infusion on PS and POP incidence, and no significant treatment effects were observed. Understanding how the sow vaginal microbiota is affected by antibiotics and their route of administration may be helpful in this context to better understand sow reproductive health. Alternatively, a focus on increasing bacteria associated with PS1 sows could be another focus for a mitigation strategy. Regardless, defining a healthy vaginal microbial population during late gestation in sows is an essential step in understanding how to combat dysbiosis.

Despite the lack of intravaginal treatment effect on POP risk and outcome in sows, this study did observe an interesting result concerning the relationship between litter characteristics and presumed risk of POP. In the current study, an increase in total born was observed in PS2 compared to PS1 assigned sows, likely influencing the overall effect of total born on PS. The lack of significance observed in total born between PS1 and PS3 sows could be due to the small sample size (*n* = 38) of litter data collected from sows assigned PS3. With a larger sample size, the numerical increase in total piglets born observed in PS3 compared with PS1 sows in the current study may have reached statistical significance. Previously, a decrease in number of live born piglets has been observed in sows that experience POP, along with an increased number of stillborn piglets, with no difference in number of total born (Ross 2019). When evaluating BMD as a POP mitigation strategy only the PS3 assigned sows in the treated group had fewer stillborn piglets when compared to PS3 sows in the control group (Studer et al. 2026). These results are similar to the current study in that stillborn piglets were higher in sows at elevated risk for POP (ie PS3) with a decrease in number born alive. While the current study did not observe a treatment effect on litter characteristics by PS, the observation that stillbirths are significantly influenced by PS suggests that if an effective way to reduce PS is identified, the potential to decrease stillborn piglets and increase the number born alive exists. Understandably, increased stillborn piglets would be associated with decreased live born piglets as these numbers are inversely correlated. The higher incidence of stillborn piglets in PS3 assigned sows could potentially be related to the increased swelling of the perineal region observed, leading to farrowing difficulties and increased piglet death. Additionally, it has previously been observed that farrowing duration and the number of stillborn piglets are directly correlated, with increased farrowing duration leading to an increase in stillborn piglets (Zaleski and Hacker 1993; Van Dijk et al. 2005). Interestingly, several studies have observed correlations between an increase in total born pigs and increased stillborn piglets (Van Dijk et al. 2005; Gourley et al. 2020). The current study observed the same effect and litters with an increased total born also had increased stillborn piglets. Presumably, increased litter sizes could relate to increased farrowing durations adding to the effect of increased stillborn piglets. Increased stillborn rates have been observed in piglets born later in the birth order from sows that had larger litter sizes (Gourley et al. 2020). This supports the above-mentioned theory of increased risk for fetal asphyxia in larger litters leading to increased stillborn piglets. The current study has a smaller sample size of litter data collected from PS3 assigned sows (*n* = 38) compared to PS1 assigned sows (*n* = 489) due to the higher incidence of POP in PS3 sows, which often occurs before or during farrowing. Therefore, litter data for sows that experience POP is typically incomplete. Farrowing duration and litter characteristic effects on piglet survival should be further studied in relation to POP risk in sows to better understand the interrelationships of litter size, PS and POP outcome.

In conclusion, vaginal infusions of ampicillin during late gestation did not affect PS or POP incidence in sows. However, litter characteristics differed between sows assigned various PS and should be further evaluated in the future, as PS may also have predictive value to enable better management strategies to support sows expected to farrow larger litters to maximize piglet livability. Further research on developing mitigation strategies for POP is warranted, and the relationship between PS and litter characteristics should be further investigated, as litter size may be an associated factor influencing the incidence of POP in some conditions.

## Funding

This project was supported by the National Pork Board and the Foundation for Food and Agriculture Research.

## List of abbreviations

BV: Bacterial vaginosis
CON: Control
ISU: Iowa State University
LBP: LPS binding protein
LPS: Lipopolysaccharide
PID: Pelvic inflammatory disease
POP: Pelvic organ prolapse
PS: Perineal score
TP1: Timepoint 1
TP2: Timepoint 2
TRT: Treatment
